# Cell-to-cell transmission of retroviruses: Innate immunity and interferon-induced restriction factors

**DOI:** 10.1016/j.virol.2010.12.031

**Published:** 2011-03-15

**Authors:** Clare Jolly

**Affiliations:** Wohl Virion Centre and MRC Centre for Medical Molecular Virology, Division of Infection and Immunity, University College London, W1T 4JF, UK

**Keywords:** HIV, Retrovirus, Virological synapse, Restriction factor, Innate immunity, Interferon, Tetherin, BST-2, Vpu

## Abstract

It has been known for some time that retroviruses can disseminate between immune cells either by conventional cell-free transmission or by directed cell-to-cell spread. Over the past few years there has been increasing interest in how retroviruses may use cell-to-cell spread to promote more rapid infection kinetics and circumvent humoral immunity. Effective humoral immune responses are intimately linked with innate immunity and the interplay between retroviruses and innate immunity is a rapidly expanding area of research that has been advanced considerably by the identification of cellular restriction factors that provide barriers to retroviral infection. The effect of innate immunity and restriction factors on retroviral cell-to-cell spread has been comparatively little studied; however recent work suggests this maybe changing. Here I will review some recent advances in what is a budding area of retroviral research.

## Cell-to-cell spread of retroviruses

Retroviruses are a diverse family of enveloped RNA viruses that encompass a number of medically important human pathogens including the Human Immunodeficiency Virus (HIV), which alone has accounted for approximately 25 million deaths worldwide. Over the past decade huge scientific and medical endeavour has been focussed towards understanding the biology of viral pathogenesis and transmission between and within hosts. Like a number of other mammalian viruses, retroviruses can disseminate between susceptible cells either by cell-free infection or by direct cell-to-cell spread (reviewed in (Sattentau, 2008)). Retroviruses spread directly between cells by taking advantage of their immunotropic properties to infect CD4^+^ T cells, macrophages and dendritic cells that inherently form intimate, dynamic and transient contacts (Jolly and Sattentau, 2004). In this way, retroviruses can co-opt specialized properties of immune cells that normally operate during intercellular communication such as antigen presentation and T cell activation to promote their dissemination between cells.

Direct cell-to-cell spread of the human retroviruses HIV type-1 (HIV-1) and HTLV-1 (Human T-lymphotropic Virus Type-1) predominantly takes place at specialized contact-induced structures known as virological synapses (VS) that act as “hot-spots” for virus transmission (Igakura et al., 2003; Jolly et al., 2004; Jolly and Sattentau, 2002; McDonald et al., 2003). VS were so named because of their resemblance to immunological synapses (IS) and the term VS was coined to describe a specific membrane receptor architecture that evolves following intimate contact between a HIV-infected T cell and an uninfected target T cell (Jolly and Sattentau, 2002). Cell-to-cell spread of HIV-1 at synapses is a generalized feature of viral dissemination and VS have been described between infected and uninfected CD4^+^ T cells (Jolly et al., 2004), between macrophages and CD4^+^ T cells (Gousset et al., 2008; Groot et al., 2008) and between virus-exposed dendritic cells and CD4^+^ T cells (McDonald et al., 2003). This phenomenon is not restricted to HIV-1, and one of the first VS described was that of HTLV-1 (Igakura et al., 2003). Longer-range intercellular transmission of HIV-1 between T cells has also been observed along cellular projections known as membrane nanotubes (Sowinski et al., 2008), while the related retrovirus murine leukeamia virus (MLV) utilizes virus-induced filopodia for efficient dissemination (Sherer et al., 2007). The relative contribution of cell-to-cell spread at VS, membrane nanotubes and via cell-free infection is difficult to quantify, but *in vitro* culture systems have demonstrated that cell-cell spread is the predominant mode of HIV-1 dissemination and this is mostly via VS (Sourisseau et al., 2007). At present there is considerable effort towards delineating retroviral protein trafficking in infected cells during cell-to-cell spread and understanding the molecular regulators of transmission in both donor and target cells (Arrighi et al., 2004; Chen et al., 2007; Gousset et al., 2008; Groot et al., 2008; Hubner et al., 2009; Rudnicka et al., 2009; Turville et al., 2004; Igakura et al., 2003; McDonald et al., 2003; Barnard et al., 2005; Jolly et al., 2004, 2007a,b; Jolly and Sattentau, 2005, 2007; Llewellyn et al., 2010; Nejmeddine et al., 2005, 2009) and readers are directed to a recent series of comprehensive reviews that consider this in detail (Feldmann and Schwartz, 2010; Jolly, 2010; McDonald, 2010; Mothes et al., 2010; Nejmeddine and Bangham, 2010; Sattentau, 2010; Waki and Freed, 2010).

In the context of viral pathogenesis, direct cell-to-cell transmission is likely to confer a number of advantages for retrovirus compared to classical cell-free infection. Firstly, cell-to-cell spread increases infection kinetics by directing virus assembly and budding to sites of cell-to-cell contact and may be one or more orders of magnitude more efficient than equivalent cell-free infection (Dimitrov et al., 1993; Mazurov et al., 2010; Chen et al., 2007; Martin et al., 2010; Sato et al., 1992; Sourisseau et al., 2007). This is achieved by obviating the rate-limiting step of extracellular diffusion that is required of cell-free virus to find a susceptible target cell. Furthermore, polarizing virus budding towards sites of cell-to-cell contact at which viral entry receptors are clustered increases the number of potentially productive transmission events and increases the likelihood of productive infection.

Secondly, it has been hypothesised that cell-to-cell spread of retroviruses could provide a replicative advantage to the virus by limiting exposure of particles to neutralizing antibodies (Martin and Sattentau, 2009). It has generally been assumed that cell-to-cell spread of retroviruses at VS might allow escape from neutralizing antibodies either by limiting the window of opportunity for antibody to engage viral antigens, or by providing a relatively protected domain at cell-to-cell interfaces that could physically exclude the relative bulk of antibodies from gaining access to virions before they attach and enter to target cells. Whether VS protect retroviruses from humoral immunity is still unclear and there are conflicting reports on this in the literature (Chen et al., 2007; Ganesh et al., 2004; Martin et al., 2010; Massanella et al., 2009). Possible explanations for disparate results have been considered elsewhere (Sattentau, 2010) and so will not be elaborated in detail here.

Humoral immunity to human retroviruses such as HIV-1, the causative agent of Acquired Immune Deficiency Syndrome is of particular interest within the context of cell-to-cell spread because of the implications of immune evasion for vaccine design and viral pathogenesis. The innate immune response is intimately linked to the generation of an effective adaptive immune response. Thus retroviral-induced innate immune responses may have a direct impact on cell-to-cell transmission but may also modulate adaptive immunity and thereby control of viral infection. The role of innate immunity during cell-to-cell spread of retroviruses has only recently been explored; however, it is increasingly apparent that harnessing innate immunity might provide a crucial opportunity to tackle HIV-1 at some of the earliest steps of infection, and that the interplay between HIV-1 and innate immunity has important implications for disease pathogenesis (Borrow et al., 2010). In the context of HIV-1 cell-to-cell spread the balance between viral suppression and enhancement by innate immune responses is intriguing, although relatively little studied. Here I will discuss some recent insights into cell-to-cell spread and innate immunity and consider how the interplay between HIV-1 and innate effectors may modulate cell-to-cell dissemination. I will focus predominantly on HIV-1, but it is likely that some aspects of innate immunity and HIV-1 will be applicable to other retroviruses.

## Recognition of HIV-1 by innate immune receptors during cell-to-cell spread

At the earliest time points after infection, before adaptive immunity has been activated, the innate immune system provides the first line of antiviral defences and alerts the wider immune system of challenge. An important feature of innate immunity that facilitates such rapid response is the recognition of generalized pathogen-associated molecular patterns (PAMPS). This is mediated by via a range of pattern recognition receptors (PRR) including C-type lectins (CLR), Toll-like receptor (TLRs) and cytosolic sensors such as NOD-like receptors and the retinoid acid-inducible gene (RIG) like receptors RIG-I and MDA5. Recognition of ubiquitous microbial patterns leads to signal transduction, activation of the transcription factors such as NF-κB, mitogen-activated protein kinase (MAPK) and interferon regulatory factor (IRFs), and culminates in secretion of proinflammatory and immunomodulatory cytokines such as type-1 interferons (interferon-α and interferon-β). TLRs are located at the cell surface or in endocytic compartments and collectively recognize a range of viral and bacterial ligands including hydrophobic molecules, glycoproteins, bacterial cell wall components and nucleic acid, the latter being a particularly potent activator. To date, 10 different TLRs have been identified in humans. In addition, other receptors such as C-type lectins and scavenger receptors on cell surfaces can act as TLR coreceptors and bind to microbes via PAMPs which culminates in a signaling cascade that alerts the wider immune system of danger. In the context of HIV-1, a number of steps in the viral life cycle have been shown to activate immunity via PRR recognition including attachment of the HIV-1 envelope glycoprotein (Env) subunit gp120 by the C-type lectin DC-SIGN (Dendritic Cell-Specific Intercellular adhesion molecule-3-Grabbing Non-integrin) (Gringhuis et al., 2010); TLR7/8-mediated detection of HIV-1 RNA (Beignon et al., 2005; Heil et al., 2004; Meier et al., 2007) and more recently the identification of an intrinsic dendritic cell sensor that detects the interaction between newly synthesized HIV-1 capsid and cylophilin A and activates the transcription factor IRF3 (Manel et al., 2010). Interestingly, cell-free HIV-1 infection may escape innate immune detection in some situations and it has been proposed that that macrophages lack a functional PRR for HIV-1 therefore attenuating NF-κB and IRF3 activation and type-1 interferon induction (Noursadeghi et al., 2009; Tsang et al., 2009).

So far, most studies examining innate immune recognition of HIV-1 have utilized cell-free virus or viral constituents, and characterized their effects on dendritic cells and macrophages. Therefore, it is unclear whether infection during cell-to-cell contact will trigger the same innate immune sensors as cell-free infection or whether it bypasses or activates different checkpoints for innate immune activation. It is reasonable to assume that infection may have diverse consequences for the cell depending upon whether viral transmission was mediated by cell-free or cell-to-cell spread. For example, it has been suggested that HIV-1 entry during cell-to-cell contact may involve endocytosis (Blanco et al., 2004; Chen et al., 2007; Hubner et al., 2009) rather than fusion at the plasma membrane. Although the concept of productive infection via endocytosis remains controversial, if it is correct then the use of different modes of virus entry (e.g., fusion at the plasma membrane vs. endocytosis) may mean that viral constituents could be differentially presented or protected from innate immune receptors. Furthermore, polarization of viral egress towards target cells during cell-to-cell spread increases the amount of viral protein and nucleic acid that enters the target cell that may in turn increase viral antigen above a critical threshold to trigger an innate response. Notably, the Greene lab have very recently reported that the accumulation of abortive reverse transcription intermediates in resting CD4^+^ T cells following contact with infected cells activates proinflammatory and apoptotic host defences by the persistent exposure to cytoplasmic DNA, resulting in death of these non-productively infected cells (Doitish et al., 2010). Intriguingly, it was noted that indirect killing was dependent on close cell-to-cell contact and was mediated by transmitting HIV-1 virions but not cell-associated Env (Doitish et al., 2010). This effect could be recapitulated by spinoculation of cell-free virus onto cells, suggesting that the quantity of virus transferred to resting T cells during cell-to-cell contact results in an accumulation of defective cytoplasmic viral DNA triggering an IRF-3-dependent innate immune response by a TLR-independent mechanism (Stetson and Medzhitov, 2006). It will be informative to determine to what extent cell-to-cell spread of HIV-1 results in cell death rather than productive infection in activated and resting T cells when virus is transmitted across the different types of VS and how this is regulated. For example mRNA encoding TLR 1, 2, 3, 4 5, 7 and 9 have been detected in human CD4^+^ T cells (Holm et al., 2009; Xu et al., 2005). TLR activation can functionally result in cytokine secretion from T cells but we do not yet know if cell-to-cell spread triggers TLR recognition in CD4^+^ T cells. It is also unclear if dendritic cells and macrophages can serve as target cells rather than donor cells during cell-to-cell spread. DCs and macrophages efficiently transmit infectious HIV-1 *to* susceptible CD4^+^ T cells, but whether infected T cells can transmit virus *back* to DCs and macrophages, is unknown. This is an important question since it is this lineage of cell, rather than T lymphocytes, that dominate innate immune type-1 interferon secretion *in vivo*. It is clear that more work is needed in this area to address these and other questions.

## Modulating innate immunity to promote cell-to-cell spread of HIV-1

As the first line of the cellular innate response to infection, patrolling sentinel cells such as plasmocytoid and myeloid dendritic cells (pDC and mDC, respectively), Langherhan cells and macrophages are all poised to detect and engage foreign invaders. Paradoxically, some of these cells are also among the earliest targets for HIV-1 *in vivo.* Whether cell-to-cell spread triggers or suppresses antiviral responses, either virus-induced or coincidental, that the virus might use to its own advantage is an interesting proposition and number of recent studies have started to explore how HIV-1 may modulate innate immunity specifically during cell-to-cell spread. Mature myeloid DCs in particular are thought to play a key role in HIV-1 transmission between hosts by capturing incoming virions at mucosal surfaces and disseminating virus directly to CD4^+^ T cells by cell-to-cell transmission, thereby allowing HIV-1 to take advantage of the inherent response of activated mDCs to migrate to secondary lymphoid organs and interact intimately with T cells. DCs do not usually become productively infected with HIV-1 even *in vitro*, although they do express the appropriate HIV-1 entry receptors (CD4 and a chemokine co-receptor) and it generally held that productive infection by HIV-1 is a property of immature mDCs (Turville et al., 2004). As a consequence, dissemination of HIV-1 by mature mDCs to T cells is considered to mostly occur *in trans* - a process by which HIV-1 virions are captured by receptors expressed on the surface of mDCs and transferred directly to T cells during intimate cell-to-cell contact. This occurs by capture of HIV-1 particles by DC-SIGN present on the surface of DCs (Geijtenbeek et al., 2000) that recognise moieties on the Env subunit gp120, although other receptors can also mediate *trans*-infection (de Witte et al., 2007a; Lambert et al., 2008; Turville et al., 2001). Once the HIV-1-DC-SIGN complex is formed it may remain at the plasma membrane or become internalized into a partially protective endocytic compartment (thus avoiding complete degradation of infectious virus) and subsequently trafficked to the cell-to-cell junctions during VS formation (Geijtenbeek et al., 2000; McDonald, 2010; McDonald et al., 2003). By contrast, HIV-1 capture by Langerhans cells expressing the receptor Langerin results in degradation of virus (de Witte et al., 2007b). To date, most work has focussed on conventional mDCs and it is not clear whether pDC can also capture HIV-1 and transmit virus to T cells and this is no doubt complicated by the fact that pDC are more difficult to work with and comprise only 1% of peripheral blood mononuclear cells (PBMCs); however, there are reports that the related retrovirus HTLV-1 can infect pDCs (Colisson et al., 2010; Hishizawa et al., 2004) and so parallels to HIV-1 may exist.

DC-SIGN is a PRR and a C-type lectin that normally binds carbohydrate-containing ligands and initiates a response to foreign antigen via activating Toll-like receptors (Robinson et al., 2006). Insight into how HIV-1 may modulate signaling via DC-SIGN to promote cell-to-cell spread has come from recent studies investigating the DC-SIGN signalosome. Using gene expression profiling and phosphoproteomics, Hodges et al., observed that HIV-1 interaction with DC-SIGN triggers a signaling pathway leading to activation of LARG and increased RhoA-GTPAse activity and that this is necessary for efficient DC-T cell VS formation and cell-to-cell spread, possibly by RhoA modulation of exocytosis from DCs or regulation of actin dynamics (Hodges et al., 2007). HIV-1 induced DC-SIGN activation also synergises with TLR8 activation by HIV-1 ssRNA for recruitment of transcription factors required for full-length viral transcript synthesis under conditions where DCs do become productively infected and transmit virus to T cells *in cis* (Gringhuis et al., 2010). In these ways, HIV-1 can take advantage of binding to a PRR to direct downstream signaling events that favour cell-to-cell spread.

How does HIV-1 avoid degradation when internalized into DCs and transmit efficiently to T cells? Recent evidence suggests that a contributing factor maybe the ability of HIV-1 to down-regulate the autophagy pathway in cells. Autophagy is a specialized lysosomal degradation pathway of self-digestion that is necessary for correct antigen processing and presentation by MHC class II and for the delivery of TLR ligands to endosomes for innate immune activation (Virgin and Levine, 2009) and there is evidence that this pathway can be hijacked by viruses to promote pathogenesis (Kudchodkar and Levine, 2009). HIV-1 gp120 binding to CD4 on DCs has been shown to down-regulate autophagy in DCs by mTor activation and regulate cell-cell spread *in trans* (Blanchet et al., 2010). Inducing autophagy with rapamycin also inhibits DC-T cell transmission by virus-pulsed DCs (Blanchet et al., 2010) suggesting that regulating the autophagy pathway may play a key role in early HIV-1 spread by diverting virus from antigen processing pathways and allowing infectious virus to remain within the DC for subsequent delivery to the VS. Cell-free HIV-1 infection of macrophages also inhibits rapamycin-induced autophagy (Van Grol et al., 2010) and increases HIV-1 cell-free yield (Espert et al., 2006; Kyei et al., 2009); however whether this affects cell-to-cell spread specifically was not investigated. Moreover, HIV-1 also down-regulates autophagy in CD4^+^ T cells during productive infection. By contrast to DCs however, CD4-Env binding on T cells was found to activate autophagy, but this was overridden by virus infection although the effect on cell-to-cell spread was not elucidated (Espert et al., 2006).

In addition to potentially directly affecting infectious virus trafficking in cells, another consequence of loss of autophagosome is seen in HIV-1 exposed DCs that display altered TLR4 and TLR8 responses (Blanchet et al., 2010). The direct association of HIV-1 with DC-SIGN also has consequences for TLR4 signaling leading to increased expression of IL-6, IL-10 and IL-12 with presumed downstream effects on Th differentiation (Gringhuis et al., 2009) and possible consequences for viral dissemination. There is also evidence that HIV-1 limits DC maturation with consequences for CD4^+^ T cell proliferation, cytokine secretion and adaptive immunity (Kawamura et al., 2003). Thus it appears that the ability of HIV-1 to modify the cellular autophagy pathway in immune cells and thus avert innate and adaptive immunity may be at the heart of efficient cell-to-cell spread and dissemination of HIV-1 from mucosal surfaces, thereby allowing the virus to establish a foothold during early transmission and contributing to subsequent spread between target cells and cellular reservoirs.

## Cell-to-cell spread and interferon-induced, antiviral restriction factors

**Fig. 1:**
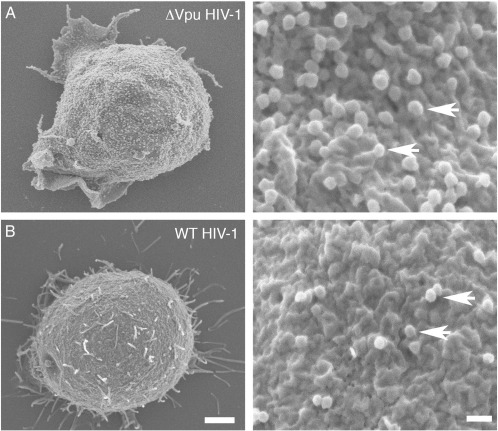
Scanning electron micrographs (SEM) showing the characteristic tetherin-mediated restriction of HIV-1 release from T cells. A) Jurkat T cells infected with Vpu-defective HIV-1 (ΔVpu- HIV-1) and B) Jurkat T cells infected with Vpu-expressing WT HIV-1 (WT HIV-1) were imaged by SEM at 7 days post-infection. Left hand panel scale bar = 500nm. Right hand panels show higher magnification of the T cell plasma membrane arrows, scale bar = 200nm. Some example virions are indicated with arrows. Note that more virions are tethered at the plasma membrane of cells infected with Vpu-defective virus (upper panels).

The induction of type-1 interferon upregulates expression of a large number of interferon inducible genes (ISGs), some of which encode proteins with direct antiviral properties. One of the most important of these within the context of retroviral dissemination is a group of proteins with potent antiviral properties known collectively as “restriction factors”. Restriction factors are cellular proteins that are constitutively expressed or induced by type-1 interferon and are able to limit viral replication by targeting specific steps of the retroviral viral life cycle (reviewed in (Wolf and Goff, 2008)) rendering cells less permissive or non-permissive to infection. In the context of human retrovirus infection three major restriction factors have now been described – APOBEC3G/F (apolipoprotein B mRNA editing complex catalytic subunit) (Sheehy et al., 2002); TRIM5 (Tripartite motif-containing protein 5) (Stremlau et al., 2004) and tetherin (also known as BST-2, CD317 and HM1.24) (Neil et al., 2008). The importance of innate restriction factors to infection is highlighted by the fact that lentiviruses contain genes encoding for accessory proteins specifically to antagonize restriction - Vif that inhibits APOBEC3-mediated cytidine deamination of viral transcripts, and in the case of HIV-1 Vpu that overcomes tetherin-mediated inhibition of nascent particle release from the plasma membrane of virus-producing cells (Fig. 1). Restriction factors are of particular interest to retroviral pathogenesis because they exert such potent inhibition of viral replication and are upregulated in some cell types by interferon, suggesting that they form part of the innate antiviral defence against viral challenge. This has raised the possibility of harnessing innate immunity and type-1 interferon induction to upregulate endogenous restriction factor expression *in vivo*, or possibly using gene-therapy to introduce restriction factors from another species that may be resistant to antagonism by viral proteins or that may have restrictive properties that do not exist in equivalent proteins from the host species. Restriction factors and inhibition of cell-free virus by retroviruses and other enveloped viruses has been a very active area of research for a number of years (reviewed in (Neil and Bieniasz, 2009; Wolf and Goff, 2008)). By contrast, less is known about whether restriction factors are inhibitory during cell-to-cell spread, although some studies are beginning to address these questions.

### TRIM5 and APOBEC

When considering the site of action of restriction factors in the context of cell-to-cell spread it is helpful to consider restriction factors in two groups, divided on the basis of which steps they target in the cell-free retroviral life cycle: those that block HIV-1 exit from the virus transmitting donor cell (e.g., tetherin and ISG15) and those that may act to restrict the early steps of virus infection (e.g., APOBEC3 and TRIM). Regarding restriction factors that target early steps (pre-integration) in the retroviral life cycle once it enters a target cell, the pertinent question is whether the restriction factor is saturable and therefore whether cell-to-cell spread may overwhelm the existing cytoplasmic pool of protein laying in wait for viral invaders. Despite the uncertainty about the mechanism of productive entry during cell-to-cell spread (endocystosis or fusion at the plasma membrane fusion), restriction factors target steps of the retroviral life cycle (uncoating, reverse transcription and integration) that are essential for successful proviral integration and must be achieved for productive infection to ensue.

Rhesus TRIM5α is a potent inhibitor of HIV-1 infection that restricts at different stages of the viral life cycle probably by promoting capsid disassembly (Chatterji et al., 2006; Stremlau et al., 2006), inhibiting reverse transcription (Stremlau et al., 2004) and preventing integration (Anderson et al., 2006; Diaz-Griffero et al., 2007; Wu et al., 2006). Notably, there is clear species specificity in TRIM5 activity and HIV-1 is largely resistant to restriction by human TRIM5 but is sensitive to restriction by TRIM5 from Old World Monkeys (Stremlau et al., 2004). This has raised the possibility of engineering human cells to express rhesus TRIM5 (rhTRIM) as a therapeutic intervention to target HIV-1. Since human TRIM5 cannot restrict HIV-1 the issue of cell-to-cell versus cell-free restriction was addressed by engineering primary human CD4^+^ T cells to express rhTRIM5 and investigating whether transmission of virus between T cells was efficiently restricted (Richardson et al., 2008). Taking this approach Richardson et al., reported that rhTRIM5 efficiently blocked cell-free infection, but not infection mediated by cell-to-cell spread. Moreover they observed that inhibition of HIV-1 spreading infection in *in vitro* culture required rhTRIM5 to be expressed in both the virus-producing cell and the target cell and rhTRIM5 was unable to inhibit cell-to-cell spread unless expressed by the majority of cells in culture. It has previously been suggested, albeit controversially, that TRIM5 can restrict the production infectious HIV-1 when expressed in the producer cell (Sakuma et al., 2007) but this is unlikely to be the mechanism of restriction of cell-to-cell transmission. It is most likely that cytoplasmic TRIM5 in target cells can be saturated by the increase in capsid that is transmitted during cell-to-cell spread and so expression of rhTRIM5 in the donor cell allows rhTRIM to also associate with capsid during virus production, thus tipping the scales in favour of restriction upon entry into target cells.

Members of the APOBEC family of restriction factors are incorporated into nascent virions in the virus-producing cell that inhibit retroviral infection in target cells by deaminating dC to dU in nascent minus-strand DNA, resulting in G-to-A hypermutation (Harris et al., 2003; Lecossier et al., 2003; Mangeat et al., 2003) thereby inhibiting reverse transcription and integration (reviewed in (Albin and Harris, 2010). This is overcome by HIV-1 Vif that prevents packaging of APOPBEC into particles during virus assembly in infected cells, in part by proteasome-dependent degradation and/or possibly by direct inhibition of encapsidation (reviewed in (Albin and Harris, 2010)). APOBEC is induced by type-1 interferon in macrophages but not activated primary T cells or T cell lines (Koning et al., 2009; Refsland et al., 2010; Stopak et al., 2007) and it has been suggested that within the target cell APOBECs are unlikely to be strong inhibitors (Goujon and Malim, 2010). To date no studies have addressed whether APOBECs restrict cell-to-cell spread as efficiently as cell-free infection, but it seems most likely that cell-to-cell spread would be similarly sensitive to APOBEC-mediated DNA editing since there is no reason why Vif should not exclude APOBEC encapsidation during *de novo* virus assembly at the VS (Hubner et al., 2009), unless the rapid and polarized assembly of virions temporally or spatially precludes efficient Vif activity for some reason. Further work is needed to fully understand how APOBECs get incorporated into nascent virus. For example, at what stage they encounter viral RNA, whether this may differ between cell-free virus production or assembly at the VS, and where Vif interacts with APOBEC in order to speculate about whether cell-to-cell spread may be similarly susceptible to APOBEC-mediated restriction in the absence of Vif, or whether some APOBEC may slip through. In addition to TRIM5 and APOBECs it is very likely there are other as yet undiscovered restriction factors that target post-entry steps of HIV-1 infection in target cells (Goujon and Malim, 2010) and further investigation of restriction of cell-to-cell spread is certainly warranted.

### Tetherin and ISG15

**Fig. 2:**
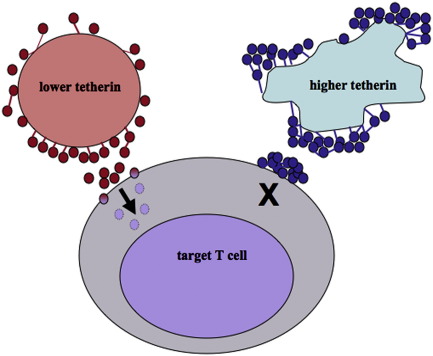
A proposed model for cell-to-cell spread of Vpu-defective HIV-1 in the presence of different amounts of unantagonized tetherin on the surface of the virus-producing cell. Under conditions where the donor cell expresses lower levels of tetherin (e.g., endogenous expression on T cells) cell-to-cell spread can occur and target T cells become productively infected. When the donor cell expresses very high levels of tetherin (e.g., epithelial cells such as HeLa cells or cells over-expressing plasmid-encoded tetherin) then virus could be transferred as unusually large, tethered aggregates that cannot fuse appropriately at the plasma membrane and productive infection would be blocked.

Evidence to date suggests that the general mechanism of assembly and budding of retroviruses from productively infected CD4^+^ T cells and macrophages at the VS is the same as that of cell-free virus release (Gousset et al., 2008; Groot et al., 2008; Hubner et al., 2009; Jolly et al., 2004; Martin et al., 2010; Mazurov et al., 2010). Live cell imaging has previously revealed *de novo* assembly of HIV-1 and MLV with preferential viral assembly at the contact site (Hubner et al., 2009; Jin et al., 2009). Retroviral cell-to-cell spread may also occur without significant *de novo* assembly via the transfer of infectious virions that have budded through the plasma membrane but remain associated with the cell surface by interactions with cellular proteins (Pais-Correia et al., 2010; Sherer et al., 2010). It can postulated then that restriction factors that target late steps in virus production in donor cells might be similarly active at inhibiting viral egress during either cell-free transmission or direct cell-to-cell spread. Conversely cell-to-cell spread may saturate a restriction factor if it was not associated with the VS at the right time and in sufficient quantity. Moreover, different mechanisms of cell-to-cell spread (e.g., *de novo* virus production at cell-to-cell contacts versus lateral movement of budding virus from distal membrane domains towards the VS) may be susceptible to restriction while others may be less so. Two studies have recently addressed this question and considered whether cell-to-cell spread of HIV-1 between T cells is sensitive or resistant to restriction by tetherin, with apparently conflicting results. Casartelli et al. reported that tetherin inhibited cell-to-cell spread of HIV-1 and that tetherin blocked productive transmission by reducing viral fusigenicity and thus infectivity. Unusually large aggregates of virus were transferred from tetherin-expressing HeLa donor cells to target cells and this virus was less able to initiate productive infection (Casartelli et al., 2010). By contrast, Jolly et al. found that productive cell-to-cell spread of HIV-1 between T cells was not restricted by endogenous tetherin expressed on donor T cells and infectious virus was transmitted across the T cell VS resulting in productive infection (Jolly et al., 2010). The observation that Vpu-defective virus is transmitted as efficiently, if not more so than WT virus is in agreement with a number of previous studies (Gummuluru et al., 2000; Klimkait et al., 1990; Schubert et al., 1995; Sourisseau et al., 2007; Strebel et al., 1989; Terwilliger et al., 1989; Yao et al., 1993). Possible reasons for the differences observed between our study and Casartelli et al. may not be immediately obvious since both groups agree that tetherin is present at the T cell VS and that synapses appear to be form normally in the presence of tetherin on the virus-producing cell (Casartelli et al., 2010; Jolly et al., 2010). In both studies, cell-to-cell spread of HIV-1 was interrogated in the presence or absence of Vpu using Vpu-expressing or non-expressing virus: Vpu-defective virus does not antagonize tetherin and results in the well-characterized budding defect where nascent, proteolytically matured virions remain attached to the surface of the virus-producing cell by membrane tethers (Neil et al., 2008; Strebel et al., 1989; Van Damme et al., 2008). In the presence of Vpu, tetherin activity is abrogated and the protein is degraded via the proteasome (Goffinet et al., 2010a; Mangeat et al., 2009; Van Damme et al., 2008) and/or lysosome (Douglas et al., 2009; Iwabu et al., 2009; Mitchell et al., 2009), but whether this results in global down-regulation of tetherin from the plasma membrane, or simple exclusion of tetherin from membrane regions that prevent association with viral proteins is unclear. The findings of Casartelli et al., and Jolly et al., may be somewhat reconciled by considering the type of the donor cells used to examine cell-to-cell spread and the chronicity of the infection, since the experimental assays were broadly similar. It may be that in the presence of lower levels of tetherin, such as is endogenously expressed on T cells (used by (Jolly et al., 2010)), productive cell-to-cell spread can take place without effective restriction (Fig. 2). Tetherin expressed on the surface of T cells would be sufficient to retain virions at the cell surface and in this way mature infectious virus attached to the plasma membrane by tetherin would be poised to engage CD4 on target cells facilitating VS and polysynapse formation and more rapid cell-to-cell spread as we observed with Vpu-defective HIV-1 (Jolly et al., 2010). Notably, we did not detect any loss of viral infectivity of virus produced from T cells when Vpu was absent and tetherin was unantagonized (Jolly et al., 2010). By contrast HeLa cells or 293T cells transfected with plasmid-encoding tetherin express higher levels of tetherin at the cell surface (Miyagi et al., 2009; Neil et al., 2007, 2008; Van Damme et al., 2008). When these cells were used as donor cells productive cell-to-cell spread was inhibited due to the formation of unusually large viral aggregates. Viral aggregates were transferred to target cells but remained stuck at the cell surface and virus did not appear to fuse appropriately at the plasma membrane, leading to reduced infectivity (Casartelli et al., 2010) (Fig. 2). This effect may be explained by large amounts of tetherin being incorporated into virions. A recent report similarly observed an apparent reduction HIV-1 infectivity in the presence of tetherin (Zhang and Liang, 2010); however in this study virus was also produced from epithelial cells co-transfected with tetherin-encoding plasmid therefore resulting in higher tetherin expression that would be expected on T cells. It should be noted that variations in tetherin expression have also been reported between different T cell lines (Miyagi et al., 2009) and this too may account for some of the discordant results in cell-to-cell spread when T cells were used. It will be interesting to see if future studies probe the infectivity of HIV-1 produced from infected T cells and if so, whether they observe reduced infectivity or not in the presence of tetherin.

Considering the chronicity of the infected cells, we generally used cells later after initial infection in order to maximise the percentage of infected cells used in our assays to obviate any effect of virus spreading within the donor population in co-culture assays. It is possible that allowing the infection to proceed for longer in T cells could also increase the amount of proteolytically mature, infectious virus tethered at the cell surface thus enhancing transmission of Vpu-defective virus. Moreover, it is possible that longer incubations may provide opportunity for cell-to-cell contacts to occur that could induce budding but keep nascent virus trapped at the cell surface, there by forming structures similar to tetherin-containing viral biofilms that have been shown to facilitate cell-to-cell spread of HTLV-1 at VS (Pais-Correia et al., 2010). Using cells at earlier times post-infection may result in less tethered virus accumulating at the plasma membrane of T cells infected with Vpu-defective virus resulting in reduced virus transfer. It may also be speculated that *de novo* virus assembly at the VS may result in a shift in the balance of immature vs. mature virions being transferred by cell-to-cell spread in favour of non-infectious, immature virus.

It is interesting to note that neither HTLV-1 nor MLV encode known tetherin antagonist but disseminate predominantly via cell-to-cell spread. Recent evidence suggests that tetherin can partly reduce MLV transmission in mouse cells by an as yet unknown mechanism (Goffinet et al., 2010b) but whether this is due to unidentified antagonism by an MLV protein or another mechanism is unclear. Thus it seems possible that HIV-1 may use different mechanisms under certain conditions and that retroviruses may also be able to use tetherin to its advantage in some situations. Moreover, it is also possible that different cell-to-cell interactions (e.g., DC-T cell or macrophage-T cell) may shift the balance towards transmission or restriction by tetherin during cell-to-cell spread. Notably, T cell-to- T cell spread is less sensitive to interferon-mediated inhibition than cell-free infection suggesting that cell-to-cell transmission can partially overcome interferon-induced blocks to transmission (Vendrame et al., 2009). It is tempting to speculate that differences in cell-to-cell versus cell-free spread in the presence or absence of interferon may account for the variable results seen using interferon-based therapy (Frissen et al., 1997; Hatzakis et al., 2001; Hulton et al., 1992; Kaiser et al., 1992; Landau et al., 2000; Rivero et al., 1997; Skillman et al., 1996; Sperber et al., 1993). Clearly more work is needed to clarify this area and to more completely delineate the different possible mechanism of HIV-1 cell-to-cell spread and how tetherin may fit into this.

Other interferon inducible cellular proteins can limit HIV-1 release such as ISG15, a ubiquitin-like protein that inhibits ubiquitination of Gag and Tsg101 which arrests HIV-1 budding at a late stage (Okumura et al., 2006) but whether ISG15 is similarly active against cell-to-cell spread in unclear. Late-budding defects involving ESCRT are characterized by the accumulation of immature virus at the cells surface and immature HIV-1 is not infectious, thus is it likely that ISG15 would interfere with cell-to-cell spread.

## Concluding remarks

Innate immune activation and interferon secretion following exposure of cells to retroviruses such as HIV-1 has important consequences for immune regulation and is a double-edged sword during HIV-1 infection (Chang and Altfeld, 2010). In the short term, innate immune activation is necessary for recruitment of effector cells and initiating adaptive immunity; however the recruitment of target T cells and macrophages, for example to sites of virus infection, and subsequent T cell activation increases the local pool of susceptible target cells able to support robust viral replication. Moreover, chronic immune activation will result in the loss of CD4^+^ T cells, homeostatic inbalance and T cell exhaustion, increasing the risk of disease progression and opportunistic infections. How cell-to-cell spread is modulated by innate immunity and how viral dissemination maybe counteracted by innate recognition is an emerging area of research and further work is clearly needed to address the molecular mechanisms of cell-to-cell spread in the context of innate immunity and the role of interferon-induced restriction factors in this. Early insights into what is a budding area of research, are turning out to be intriguing and probably have consequences for therapeutic intervention and future efforts to tackle HIV-1/AIDS.
